# Ghrelin Alleviates Endoplasmic Reticulum Stress in MC3T3E1 Cells by Inhibiting AMPK Phosphorylation

**DOI:** 10.1155/2021/9940355

**Published:** 2021-10-11

**Authors:** Xue Lv, Qianping Zhang, Bingfei Cheng, Ying Xin, Jun Wang, Juanjuan Li, Chengqian Li, Nailong Yang

**Affiliations:** ^1^Department of Pathology and Pathophysiology, School of Medicine, Zhejiang University, Hangzhou, Zhejiang 310058, China; ^2^Department of Endocrinology and Metabolism, Dezhou Municipal Hospital, Dezhou, Shandong 253000, China; ^3^Department of Endocrinology and Metabolism, The Affiliated Hospital of Qingdao University, Qingdao, Shandong 266555, China

## Abstract

Ghrelin is a gastric endocrine peptide that has been found to be involved in the process of energy homeostasis and bone physiology in recent years. To explore the effects of ghrelin on endoplasmic reticulum stress (ERS) in MC3T3E1 cells and its possible mechanism, an ERS model was induced by tunicamycin (TM) in the osteoblast line MC3T3E1. TM at 1.5 *μ*g/mL was selected as the experimental concentration found by CCK8 assay. Through the determination of apoptosis, reactive oxygen species production, and endoplasmic reticulum stress-related gene expression, we found that ERS induced by TM can be relieved by ghrelin in a concentration-dependent manner (*P* < 0.001). Compared with the TM group, ghrelin reduced the expression of ERS-related marker genes induced by TM. Compared with the GSK621 + TM group without ghrelin pretreatment, the mRNA expression of genes in the ghrelin pretreatment group decreased significantly (*P* < 0.001). The results of protein analysis showed that the levels of BIP, p-AMPK, and cleaved-caspase3 in the TM group increased significantly, while the levels decreased after ghrelin pretreatment. In group GSK621 + TM compared with group GSK621 + ghrelin+TM, ghrelin pretreatment significantly reduced the level of p-AMPK, which is consistent with the trend of the ERS-related proteins BIP and cleaved-caspase3. In conclusion, ghrelin alleviates the ERS induced by TM in a concentration-dependent manner and may or at least partly alleviate the apoptosis induced by ERS in MC3T3E1 cells by inhibiting the phosphorylation of AMPK.

## 1. Introduction

As a hidden and common disease, osteoporosis is characterized by a decrease in bone mass and a change in bone microstructure. Its complications, pain and brittle fracture, seriously affect the quality of life of patients and even threaten their lives. Previous studies have shown that apoptosis induced by endoplasmic reticulum stress (ERS) in osteoblasts plays an important role [1]. ERS is an “unfolded protein reaction (UPR)” [2] in the endoplasmic reticulum caused by endogenous and exogenous factors such as heat shock, ultraviolet radiation, oxidative damage, hypoxia, and hypoglycemia. The accumulation of unfolded or misfolded proteins triggers a stress response in the endoplasmic reticulum to remove these proteins, and excessive ERS can lead to apoptosis.

Ghrelin, the endogenous ligand of growth hormone secretagogue receptor type 1a (GHSR-1a), is composed of 28 amino acids [3]. In recent years, ghrelin has been found to play a role in various aspects, such as energy homeostasis [4], cell proliferation and autophagy [5], cardiovascular disease [6], respiratory disease [7], and the immune system [8]. It was found that the incidence of osteoporosis increased significantly after subtotal gastrectomy [9], and ghrelin polypeptide decreased significantly after surgery [10], which is closely related to the pathogenesis of osteoporosis, but the mechanism has not been elucidated.

AMPK (Adenosine Monophosphate Activated Protein Kinase), a heterotrimer complex, contains a highly conserved serine/threonine kinase domain and is a key molecule in the regulation of bioenergy metabolism [11]. Previous studies have shown that when blood glucose and lipid metabolism are out of balance, the activation of AMPK is the key hub to regulate this process [12].

Based on the ERS model of osteoblasts induced by tunicamycin (TM), one of the aims of this experiment was to pretreat osteoblasts with different concentrations of ghrelin, detect the apoptosis of osteoblasts and the content of intracellular reactive oxygen species (ROS), and observe the effect of ghrelin on ERS in a concentration-dependent manner. The other purpose was to verify the expression of ERS-related marker genes and the changes in related pathway factors in different groups at the molecular level to further explain the protective effect of ghrelin on ERS in osteoblasts and its possible related pathways.

## 2. Materials and Methods

### 2.1. Major Reagents

Tunicamycin (TM) was purchased from Cayman Chemical Company (USA). Ghrelin (molecular weight: 3314.8) was synthesized by Nanjing Peptide Biology Company (CN). GSK621, purchased from Cayman Chemical Company, is the agonist of AMPK.

### 2.2. Cell Culture

MC3T3E1 cells (ATCC, USA) were cultured in *α*-MEM medium (Gibco, USA) containing 10% fetal bovine serum and 1% penicillin and then maintained in a humidified, 5% CO_2_ atmosphere at 37°C. The medium was refreshed every two or three days, and the cells were subcultured using 0.05% trypsin with 0.01% EDTA.

### 2.3. Measurement of Cell Viability

Cell viability was evaluated by a CCK-8 (Solarbio, CN) method. MC3T3E1 cells were incubated overnight in a 5% CO_2_ incubator at 37°C at a concentration of 5∗10^4^ cells/ml and inoculated in a 96-well plate. After the cells in the 96-well plate were completely adhered to the wall, the culture medium with different concentrations of TM (0, 0.2, 0.4, 0.8, 1, 2, 4, and 8 *μ*g/mL) was replaced. After 20 hours, the CCK-8 reagent was added, the absorbance at 450 nm was measured by a microplate reader, and the IC50 of cells was calculated for subsequent experiments.

### 2.4. Assay of Annexin V-FITC/PI Apoptosis

After each group was treated with the corresponding drugs and the necessary control groups were set up, the cells were digested with 0.25% trypsin (excluding EDTA), washed twice with PBS to collect 5∗10^5^ cells, and resuspended in 500 *μ*L of binding buffer. Then, 5 *μ*L of Annexin V-FITC was added, 5 *μ*L PI (BD, USA) was added, and the mixture was incubated at room temperature in the dark for 15 min. Apoptosis was detected by flow cytometry. The upper left quadrant represents a mechanically injured cell, and the upper right quadrant is a late apoptotic cell. The lower left quadrant is a normal cell, and the lower right quadrant is an early apoptotic cell. In this experiment, the proportion of the upper right quadrant + the lower right quadrant was used as the percentage of apoptotic cells.

### 2.5. Reactive Oxygen Species Detection

After each group was treated with drugs, a negative control and a positive control were established at the same time. After 24 hours of culture, the cells were washed with warm PBS, and 10^6^ cells were harvested by trypsin. Then, the cells were incubated in the same volume of DCFH-DA solution (10 *μ*mol/L, Sigma, USA) at 37°C for 0.5 h according to the manufacturer's protocol so that the probes were in full contact with the cells and washed with PBS to remove the redundant probes. Flow cytometry was used to detect the production of reactive oxygen species in osteoblasts.

### 2.6. Reverse Transcription-Polymerase Chain Reaction (RT–PCR) Detection of ERS-Related Gene Expression

Cells in the logarithmic growth phase were inoculated in 6-well plates (2∗10^5^ cells/well). After adhering to the wall, cells were cultured to a density of approximately 70% and then replaced with warm drug medium under different treatment conditions including GSK621 pretreatment for 3 hours, ghrelin treatment for 4 hours, and TM followed by 20 hours. Total RNA was extracted according to the instructions (ABclonal, CN), and the concentration and purity of RNA were measured by a spectrophotometer. Using RNA as a template, quantitative reverse transcription was performed converting 1 *μ*g of into cDNA. Finally, cDNA was used as a template, and specific forward primers and reverse primers of each gene were added to amplify the cDNA. The primer (Invitrogen, USA) sequences used in this study are presented in Table 1.

### 2.7. Western Blot Analysis

After inoculation, the cells were cultured in complete medium at a density of approximately 70%, and the corresponding treatments were carried out according to the groups. Western blotting was carried out according to previous research protocols, and RIPA (Solarbio, CN) was used to extract the total proteins. A BCA protein assay kit (Beyotime, CN) was used to determine the protein concentration, and the same amount of protein (30 *μ*g) was loaded into 10% SDS–PAGE gels for electrophoresis under constant pressure. The protein was then transferred to the PVDF membrane under constant current, sealed with 8% skimmed milk powder for 1.5 hours, incubated with the primary antibody (*β*-actin, AMPK, p-AMPK, BIP, and cleaved-caspase3, 1 : 1000 dilutions; CST, USA) for 12–16 hours, washed on a shaker with TBST 4 times for 10 minutes each time, and then washed with the residual secondary antibody on a shaker again with the corresponding secondary antibody (1 : 10000 dilutions, KPL, USA). The generated protein bands were exposed and visualized on a Fusion Fx machine in a dark room using ECL luminescent solution (Meilunbio, CN).

### 2.8. Statistical Analysis

All experiments were repeated three times. Data analysis and chart making were carried out using GraphPad Prism 7.0 software or SPSS 22.0. Flow J 7.6.1 software was used to analyze the apoptosis and ROS data of flow cytometry. ImageJ software was used to analyze protein quantification. The general characteristics of the data are described as the mean ± SD or mean ± SEM. Two independent samples were compared by the Student's *t* test, and multiple samples were compared by one-way analysis of variance (ANOVA) and Tukey's multiple comparisons test. *P* < 0.05 (two-sided) was considered to indicate a significant difference.

## 3. Results

### 3.1. Endoplasmic Reticulum Stress in MC3T3E1 Cells Induced by Tunicamycin

**Figure 1:**
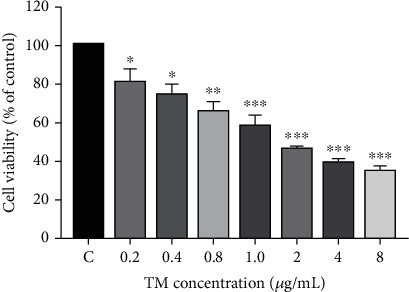
Cell viability after treatment with different concentrations of TM. After the cells were treated with different concentrations of TM (control, 0.2, 0.4, 0.8, 1, 2, 4, and 8 *μ*g/mL) for 20 h, cell viability was determined by CCK-8 kit. Data represents the mean ± SD obtained from three separate experiments. ^∗^ indicates compared with the control group, ^∗^ indicates *P* < 0.05, ^∗∗^ indicates *P* < 0.01, and ^∗∗∗^ indicates *P* < 0.001.

In Figure 1, after treating cells with different concentrations of TM for 20 hours, compared with the control group (the survival rate was set to 1), the cell vitality gradually decreased with increasing concentrations of TM. When the concentrations of TM were 0.2, 0.4, 0.8, 1.0, 2.0, 4.0, and 8.0 *μ*g/mL, the cell survival rate of each group was significantly different from that of the control group. The half cytotoxicity index was approximately 1.5 *μ*g/mL, which will be used in subsequent experiments to establish the model of endoplasmic reticulum stress in MC3T3E1 cells.

### 3.2. Ghrelin Reduced TM-Induced Apoptosis and ROS Production in MC3T3E1 Cells

#### 3.2.1. Effects of Different Concentrations of Ghrelin on the Incidence of Endoplasmic Reticulum Stress Apoptosis Induced by TM

**Figure 2:**
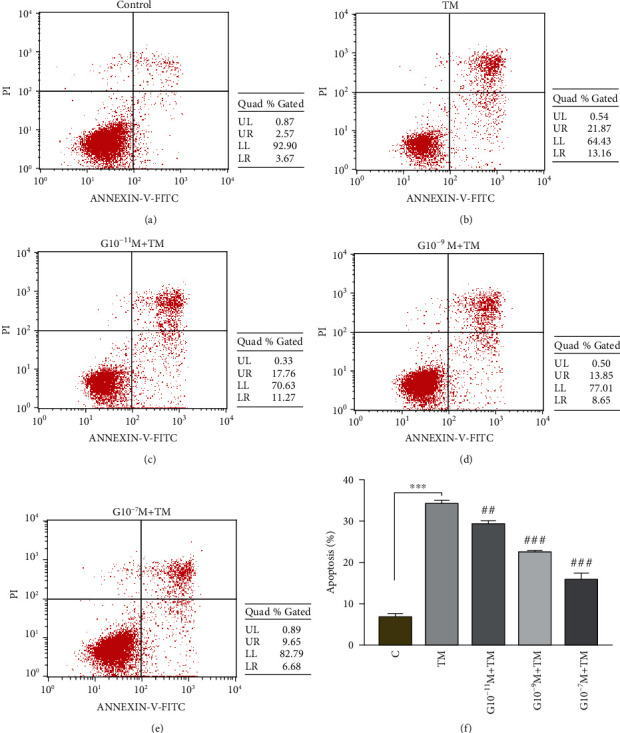
Effects of different concentrations of ghrelin on the incidence of ERS-induced apoptosis induced by TM. (a)–(e) represent the flow apoptotic patterns of the control, TM, G10^−11^M + TM, G10^−9^M + TM, and G10^−7^M + TM groups. (f) Histogram of the incidence of apoptosis in each group. Ghrelin pretreatment for 4 hours, followed by TM treatment for 20 hours. Data represents the mean ± SD obtained from three separate experiments. ^∗^ indicates comparison with the control group, ^∗∗∗^ indicates *P* < 0.001, ^#^ indicates comparison with the TM group, ^##^ indicates *P* < 0.01, and ^###^ indicates *P* < 0.001.

In Figure 2, compared with the control group (6.68 ± 0.90), the incidence of apoptosis in the TM group (34.04 ± 1.10) increased significantly (*P* < 0.001) after 20 hours of TM treatment, while after 4 hours of pretreatment with different concentrations of ghrelin, the apoptosis rate decreased significantly with the increase of the ghrelin concentration, and the *P* value of the G10^−11^M + TM (29.3 ± 0.64), G10^−9^M + TM (22.5 ± 0.26), and G10^−7^M + TM (15.6 ± 1.90) groups compared with the TM group were 0.003, <0.001, and <0.001, respectively. Ghrelin has a concentration-dependent protective effect on the TM-induced apoptosis of MC3T3E1 cells.

#### 3.2.2. Effects of Different Concentrations of Ghrelin on ROS Production Induced by TM

**Figure 3:**
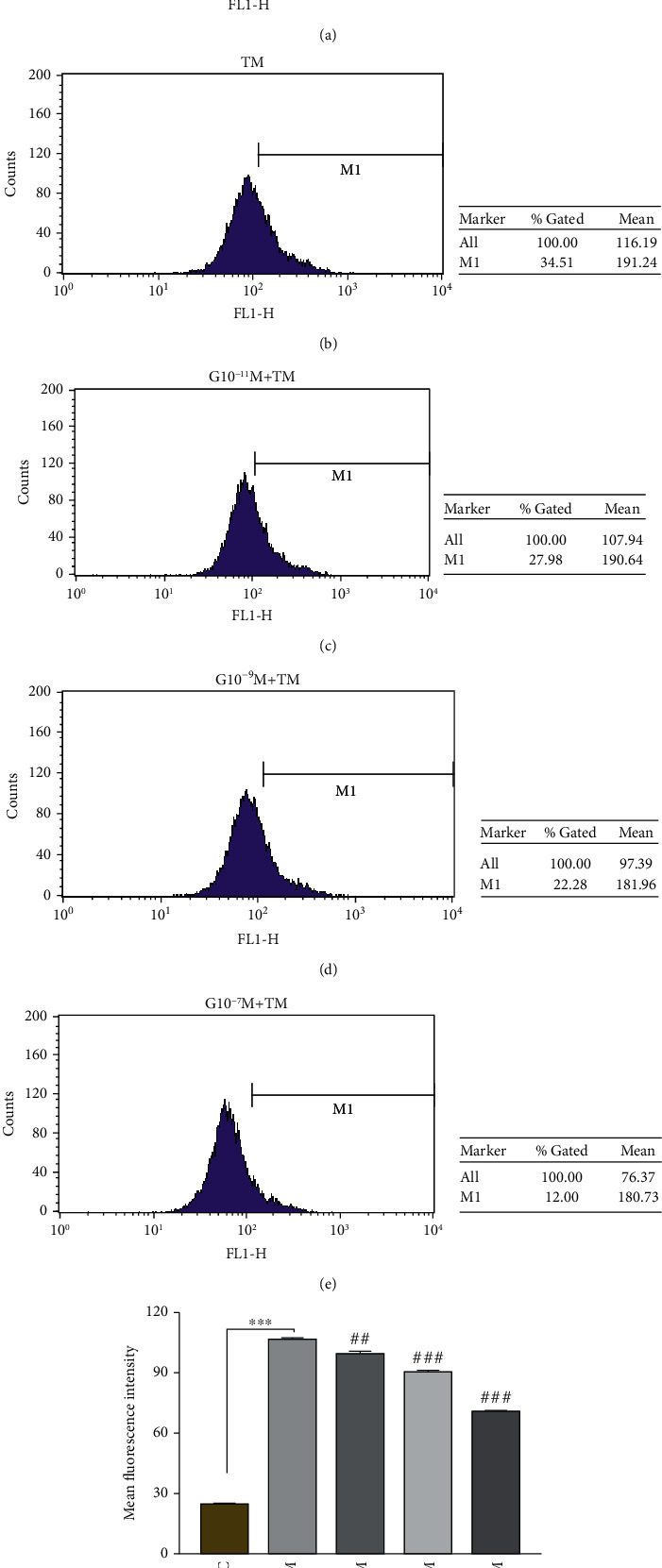
Effects of different concentrations of ghrelin on ROS production induced by TM. Determination of the fluorescence intensity of intracellular ROS induced by TM which was pretreated with different concentrations of ghrelin by a DCFH-DA fluorescence probe (flow cytometry). (a)–(e) represent the reactive oxygen flow diagram of the control, TM, G10^−11^M + TM, G10^−9^M + TM, and G10^−7^M + TM groups. (f) Histogram of the average fluorescence intensity of ROS in each group. Ghrelin pretreatment for 4 hours, followed by TM (1.5 *μ*g/mL) treatment for 20 hours. Data represents the mean ± SD obtained from three separate experiments. ^∗^ indicates comparison with the control group, ^∗∗∗^ indicates *P* < 0.001, ^#^ indicates comparison with the TM group, ^##^ indicates *P* < 0.01, ^###^ indicates *P* < 0.001.

The results of Figure 3 show that the mean fluorescence intensity of ROS in the TM group was significantly higher than that in the control group (*P* < 0.001). The fluorescence intensities of the experimental group pretreated with different concentrations of ghrelin for 4 hours were G10^−11^M + TM (107.2 ± 1.5), G10^−9^M + TM (97.6 ± 0.9), and G10^−7^M + TM (77.0 ± 0.6), which were significantly different from those treated with TM alone for 20 hours, with *P* values of 0.002, <0.001, and <0.001, respectively. In addition, ghrelin had a concentration-dependent effect on ROS production induced by TM, and the ROS content decreased with increasing pretreatment ghrelin concentration, which is consistent with the above apoptosis results.

### 3.3. Ghrelin Reduces TM-Induced Apoptosis and ROS Stress by Inhibiting AMPK Phosphorylation

#### 3.3.1. Incidence of ERS Apoptosis among Different Groups Related to TM

**Figure 4:**
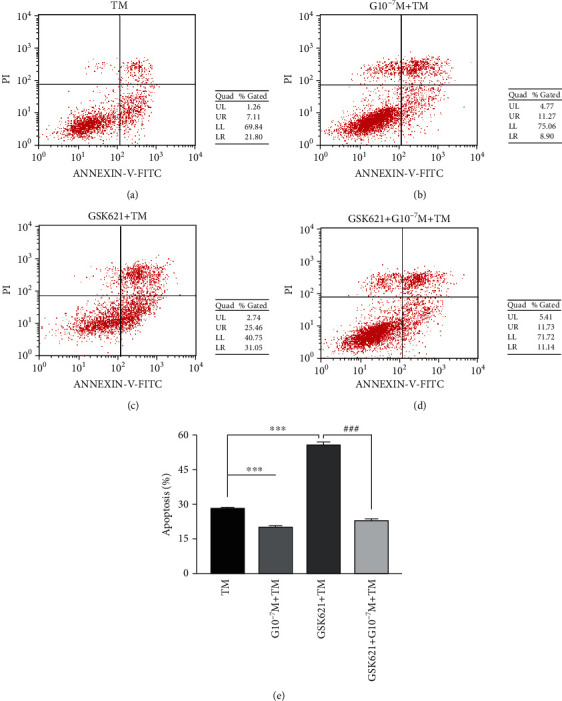
Incidence of apoptosis related to endoplasmic reticulum stress in different groups associated with TM. (a)–(d) represent the flow apoptotic patterns of the TM, G10^−7^M + TM, GSK621 + TM, and GSK621 + G10^−7^M + TM groups, respectively. (e) Histogram of the incidence of apoptosis in each group. GSK621 (10 *μ*mol/L) pretreated for 3 h, and then ghrelin (10^−7^ mol/L) was pretreated for 4 h, followed by TM (1.5 *μ*g/mL) treatment for 20 hours. Data represents the mean ± SD obtained from three separate experiments. ^∗^ indicates comparison with the TM group, ^∗∗∗^ indicates *P* < 0.001, ^#^ indicates comparison with the GSK621 + TM group, and ^###^ indicates *P* < 0.001.

Compared with TM (28.2 ± 0.64), the apoptosis rate of GSK621 + TM (55.5 ± 1.06) increased significantly (*P* < 0.001), but decreased significantly after ghrelin pretreatment (20.0 ± 0.49) (*P* < 0.001). Compared with the GSK621 + TM group, the apoptosis rate in the GSK621 + G10^−7^M + TM (22.9 ± 0.63) group decreased significantly (*P* < 0.001) (see Figure 4).

#### 3.3.2. Detection of Intracellular ROS Content among Different Groups Related to TM by DCFH-DA

**Figure 5:**
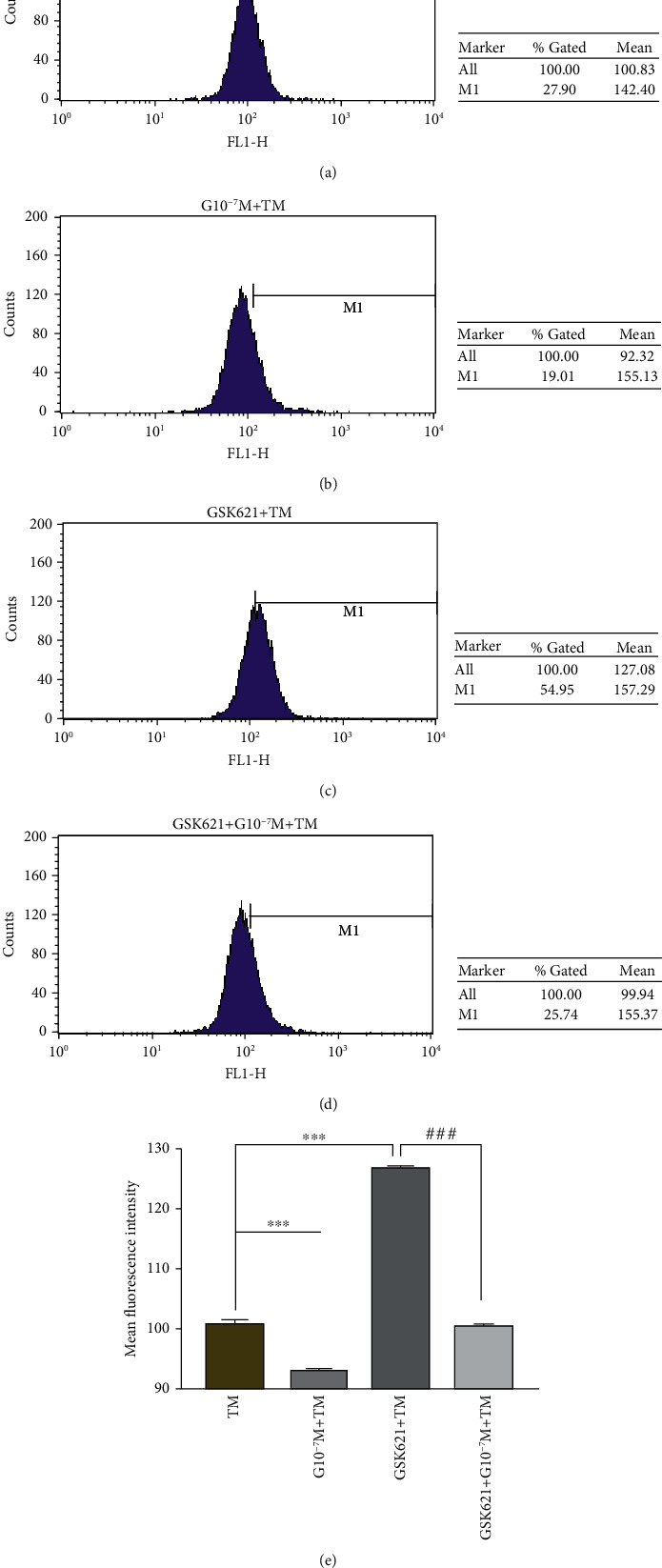
Determination of the intracellular ROS content in different groups related to TM by DCFH-DA. (a)–(d) represent the reactive oxygen flow diagrams of the TM, G10^−7^M + TM, GSK621 + TM, and GSK621 + G10^−7^M + TM groups, respectively. (e) Histogram of the average fluorescence intensity of ROS in each group. GSK621 (10 *μ*mol/L) was pretreated for 3 h, and then, ghrelin (10^−7^ mol/L) was pretreated for 4 h, followed by TM (1.5 *μ*g/mL) treatment for 20 hours. Data represent the mean ± SD obtained from three separate experiments. ^∗^ indicates comparison with the TM group, ^∗∗∗^ indicates *P* < 0.001, ^#^ indicates comparison with the GSK621 + TM group, ^###^ indicates *P* < 0.001.

The intracellular ROS content in TM + GSK621 (126.5 ± 0.57) was significantly higher than that in TM (100.6 ± 0.22), *P* < 0.001. After ghrelin pretreatment (G10^−7^M + TM, 92.8 ± 0.46), the average fluorescence intensity of ROS decreased significantly compared with that of the TM group (*P* < 0.001). Compared with that in the TM + GSK621 group, the ROS content in the GSK621 + G10^−7^M + TM group (100.3 ± 0.37) was significantly decreased (*P* < 0.001) (see Figure 5).

### 3.4. Detection of the Expression of ERS-Related Markers (BIP/GRP78, IRE1*α*, and CHOP) in Different Groups by qRT–PCR

**Figure 6:**
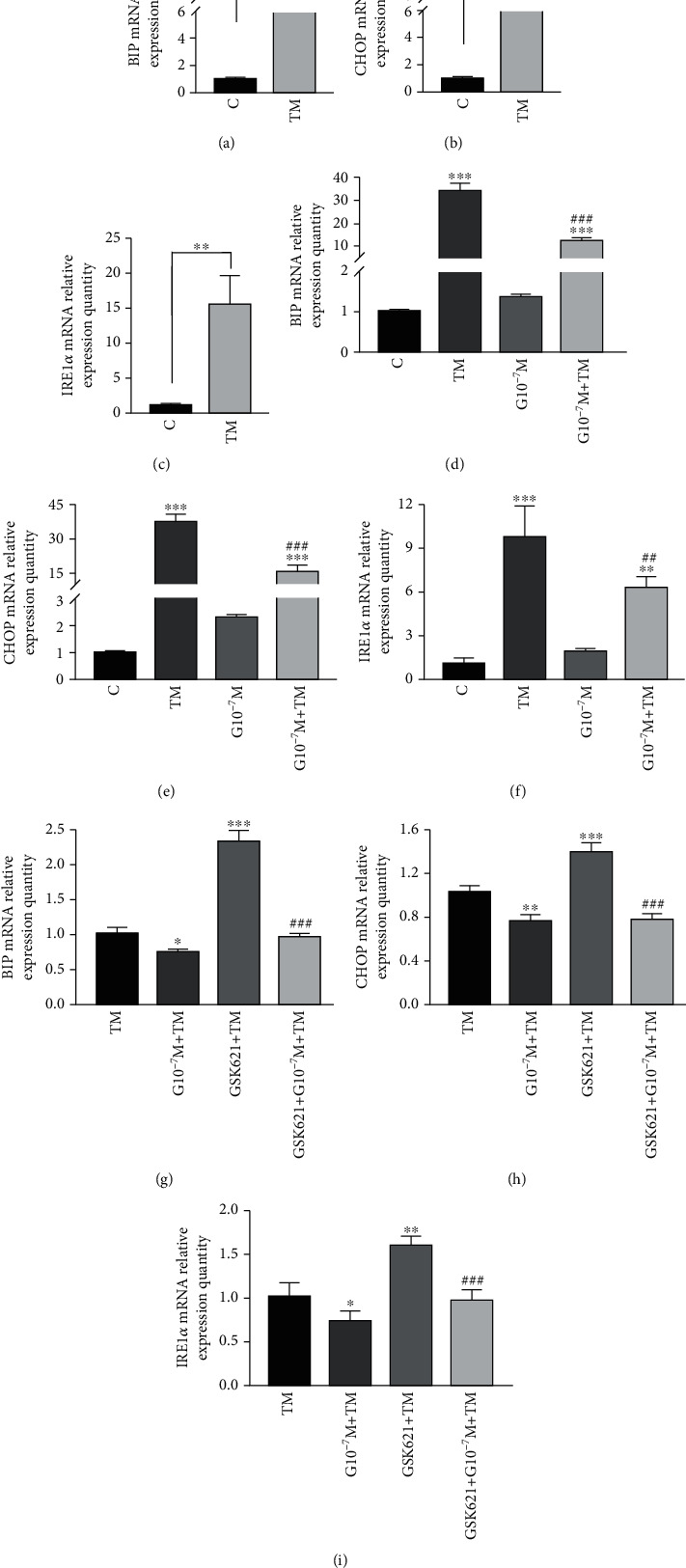
Expression of endoplasmic reticulum stress-related markers (BIP/GRP78, IRE1*α*, and CHOP) in different groups. (a)–(c) Histograms of the relative mRNA expression levels of BIP/GRP78, CHOP, and IRE1*α* after TM (1.5 *μ*g/mL) treatment for 20 hours. (d)–(f) Histograms of the relative mRNA expression levels of BIP/GRP78, CHOP, and IRE1*α* after 4 h of preconditioning with ghrelin (10^−7^ mol/L) and TM (1.5 *μ*g/mL) treatment for 20 h. (g)–(i) Histograms of the relative mRNA expression levels of BIP/GRP78, CHOP, and IRE1*α* after GSK621 (10 *μ*mol/L) pretreatment for 3 h, ghrelin (10^−7^ mol/L) pretreatment for 4 h, and TM (1.5 *μ*g/mL) treatment for 20 h. Data represents the mean ± SD obtained from three separate experiments. In (a)–(f), ^∗^ indicates comparison with the control group, ^#^ indicates comparison with the TM group, ^∗∗^ indicates *P* < 0.01, ^∗∗∗^ indicates *P* < 0.001; ^##^ indicates *P* < 0.01, ^###^ indicates *P* < 0.001. In (g)–(i), ^∗^ indicates comparison with the TM group, ^#^ indicates comparison with the GSK621 + TM group, ^∗^ means *P* < 0.05, ^∗∗^ indicates *P* < 0.01, ^∗∗∗^ indicates *P* < 0.001, and ^###^ indicates *P* < 0.001.

Figures 6(a)–6(c) show that the relative expression levels of three ERS-related marker genes in the TM group were 37.13 ± 1.51 (BIP), 23.20 ± 1.27 (CHOP), and 15.37 ± 4.16 (IRE1*α*), which were significantly higher than those in the control group (*P* < 0.01). Figures 6(d)–6(f) show that there were significant differences in the three genes among the four groups (*P* < 0.05).

In Figure 6(d), the relative expression of BIP among the groups is as follows: TM (33.86 ± 2.91) vs. C (1.00 ± 0.01), *P* < 0.001; TM vs. G10^−7^M + TM (12.17 ± 1.62), *P* < 0.001, and C vs. G10^−7^M + TM, *P* < 0.001. The relative expression of CHOP among groups in Figure 6(e) is as follows: TM (36.97 ± 3.63) vs. C (1.00 ± 0.07), *P* < 0.001; TM vs. G10^−7^M + TM (16.10 ± 2.33), *P* < 0.001, and C vs. G10^−7^M + TM, *P* < 0.001. In Figure 6(f), the relative expression of IRE1*α* among groups is as follows: TM (9.71 ± 2.18) vs. C (1.07 ± 0.43), *P* < 0.001; TM vs. G10^−7^M + TM (6.28 ± 0.75), *P* = 0.007, and C vs. G10^−7^M + TM, *P* = 0.001. The expression of three endoplasmic reticulum marker genes increased after TM treatment, and this phenomenon could be improved by ghrelin pretreatment for 4 hours, although there was still a certain degree of endoplasmic reticulum stress compared with the control group. In addition, BIP, CHOP, and IRE1*α* were all *P* > 0.05 between G10^−7^M vs. C, with no significant difference.

Taking the TM group as a control group, the relative expression of BIP in Figure 6(g) is as follows: compared with TM (1.00 ± 0.09), the difference *P* values of groups G10^−7^M + TM (0.75 ± 0.04) and GSK621 + TM (2.32 ± 0.16) were 0.011 and <0.001, respectively. The relative expression of CHOP in Figure 6(h) is as follows: compared with TM (1.00 ± 0.08), the difference *P* values of groups G10^−7^M + TM (0.75 ± 0.07) and GSK621 + TM (1.39 ± 0.09) were 0.002 and <0.001, respectively. The relative expression of IRE1*α* in Figure 6(i) is as follows: compared with TM (1.01 ± 0.15), the difference *P* values of groups G10^−7^M + TM (0.72 ± 0.11) and GSK621 + TM (1.59 ± 0.11) were 0.029 and 0.001, respectively. On the one hand, ghrelin alleviated the ERS induced by TM to a certain extent, while GSK621 promoted further expression of ERS-related marker genes induced by TM. In addition, the GSK621 + G10^−7^M + TM group had a significant decrease in the relative expression of three ERS-related genes compared with the GSK621 + TM group (*P* < 0.001).

### 3.5. Relative Expression of BIP, p-AMPK, and Cleaved-Caspase3 in Each Group Detected by Western Blotting

**Figure 7:**
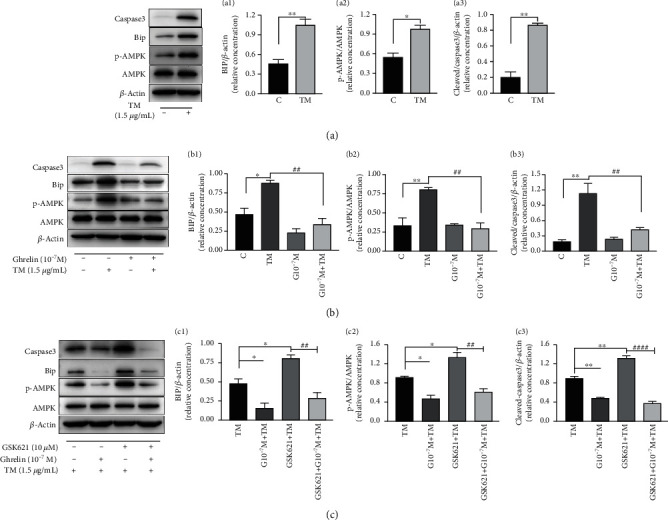
Relative expression levels of BIP, p-AMPK, and cleaved-caspase3 in different groups. BIP and cleaved-caspase3 band intensities were normalized to the *β*-actin intensity, and p-AMPK was normalized to the AMPK intensity. (a), a1-a3 are the grayscale image and histogram of the relative expression of BIP, p-AMPK, and cleaved-caspase3, respectively, after TM treatment for 20 hours. (b), b1-b3: The grayscale image and histogram of the relative expression levels of BIP, p-AMPK, and cleaved-caspase3 after 4 h of preconditioning with ghrelin (10^−7^ mol/L) and TM (1.5 *μ*g/mL) treatment for 20 h. (c), c1-c3: The grayscale image and histogram of the relative expression levels of BIP, p-AMPK, and cleaved-caspase3 after GSK621 (10 *μ*mol/L) pretreatment for 3 h, ghrelin (10^−7^ mol/L) pretreatment for 4 h, and TM (1.5 *μ*g/mL) treatment for 20 h. Data represents the mean ± SEM obtained from three separate experiments. In (a1)–(a3), 7b1-b3, ^∗^ indicates comparison with the control group, ^#^ indicates comparison with the TM group, ^∗^ indicates *P* < 0.05, ^∗∗^ indicates *P* < 0.01, ^##^ indicates *P* < 0.01; In (c1)–(c3), ^∗^ indicates comparison with the TM group, ^#^ indicates comparison with the GSK621 + TM group, ^∗^ indicates *P* < 0.05, ^##^ indicates *P* < 0.01, and ^####^ indicates *P* < 0.0001.

Figure 7(a) shows that the relative expression levels of BIP (1.03 ± 0.09 vs. 0.45 ± 0.07, *P* = 0.007), p-AMPK (0.96 ± 0.08 vs. 0.53 ± 0.07, *P* = 0.015), and cleaved-caspase3 (0.85 ± 0.03 vs. 0.20 ± 0.07, *P* = 0.001) in the TM group were significantly higher than those in the control group.

In Figure 7(b), except for the results similar to those in Figure 7(a), the expression of both proteins in the G10^−7^M + TM group pretreated by ghrelin is significantly lower than that in the TM group, including BIP: TM (0.87 ± 0.05) vs. G10^−7^M + TM (0.33 ± 0.09), *P* = 0.003, p-AMPK: TM (0.80 ± 0.04) vs. G10^−7^M + TM (0.29 ± 0.08), *P* = 0.003, and cleaved-caspase3: TM (1.12 ± 0.20) vs. G10^−7^M + TM (0.41 ± 0.05), *P* = 0.008. C vs. G10^−7^M showed no significant differences in the expression of the three types of proteins.

In Figure 7(c), compared with the TM (0.48 ± 0.07) group, BIP expression in the G10^−7^M + TM (0.15 ± 0.08) group decreased, *P* = 0.046; the expression of p-AMPK (*P* = 0.024) and cleaved-caspase3 (*P* = 0.001) showed a similar trend. After pretreatment with GSK621, an agonist of AMPK, for 3 hours, the relative expression of BIP, p-AMPK, and cleaved-caspase3 increased, with *P* values less than 0.05. In addition, comparing the relative expression of BIP with GSK621 + TM group (Figure 7(c1)), GSK621 + TM (0.80 ± 0.06) vs. GSK621 + G10^−7^M + TM (0.28 ± 0.08), *P* = 0.004. Meanwhile, comparing the relative expression of p-AMPK (Figure 7(c2)), GSK621 + TM (1.32 ± 0.12) vs. GSK621 + G10^−7^M + TM (0.60 ± 0.08), *P* = 0.001, and comparing the relative expression of cleaved-caspase3 (Figure 7(c3)), GSK621 + TM (1.31 ± 0.06) vs. GSK621 + G10^−7^M + TM (0.36 ± 0.06), *P* < 0.0001.

## 4. Discussion

In bone physiology, there is a dynamic balance between bone formation and bone absorption. Osteoporosis is a manifestation of the balance breaking, which is characterized by a change in bone microstructure and an increase in bone fragility [13], and the mechanism of which has received more attention in recent years. A large number of studies have proven that there is a close relationship between osteoporosis and endoplasmic reticulum stress [14, 15]. ERS apoptosis of osteoblasts disrupts the homeostasis between osteoblasts and osteoclasts, and the massive loss of osteoblasts leads to a decline in bone density.

Endoplasmic reticulum stress, as mentioned above, can be roughly understood as the self-protective state of the body caused by the accumulation of unfolded or misfolded proteins under the action of internal and external factors, also known as the “unfolded protein response (UPR),” which can also induce autophagy and apoptosis of cells beyond a certain range [16]. Generally, ERS has three classical signaling pathways, namely, IRE1*α* (inositol-requiring enzyme 1*α*), PERK (protein kinase RNA-like ER kinase), and ATF6 (activating transcription factor 6) [17]. BIP/GRP78, as a chaperone protein, normally combines with three factors to make it inactive, while CHOP is an apoptosis-related protein that can be activated by these three classical pathways to induce apoptosis [18]. Moreover, elevated expression of cleaved-caspase3 is an important indicator of apoptosis [19]. In addition, the production of ROS is often accompanied by the occurrence of stress, and it has also been reported that ROS are often the key factor in inducing the accumulation of unfolded proteins [20]. Studies have indicated that elevated levels of cellular H_2_O_2_ and O_2_^−^ in the endoplasmic reticulum induce cell apoptosis through lipid peroxidation and mitochondrial dysfunction and change calcium homeostasis [21]. Therefore, in this experiment, the relevant indices were selected as markers of ERS.

In this experiment, we first observed the incidence of apoptosis and the production of ROS in the ERS model pretreated with different concentrations of ghrelin on the premise of excluding the toxicity and side effects of ghrelin itself. Previous studies have shown that there is no significant difference between the ghrelin alone treatment group and the control group [22, 23]. Considering the physiological environment in the human body, the secretion of ghrelin was inhibited after eating behavior, and this effect was relieved to a great extent at 4 hours; thus, ghrelin treatment time of four hours was chosen [24]. In our results, it was obvious that ghrelin played a positive role in reducing the incidence of apoptosis and ROS production in osteoblasts, and the occurrence of apoptosis and the production of ROS decreased with increasing of concentration, which clarified the protective role of ghrelin in ERS. This is consistent with previous studies [25, 26].

Second, ghrelin significantly reduced the expression of ERS-related genes induced by TM and had a protective effect on cells. In addition, there was no significant difference in the three genes between the control group and the ghrelin treated group, which indicated that ghrelin itself had no significant effect on the production of ERS, excluding the possible drug effect of ghrelin itself, but the combination of ghrelin and TM cotreatment group compared with the control group showed that ghrelin could alleviate the ERS caused by TM on MC3T3E1 cells to a certain extent, but may not completely. Nevertheless, a large number of studies have shown that ERS has a protective effect on cells to a certain extent [27], but this limit is not yet known.

The activation of AMPK may partly depend on the increase in intracellular calcium [28]. When ERS is excessive, CHOP also promotes the release of Ca^2+^ through the IP3R (inositol 1, 4, 5, 5-triphosphate receptor) channel. The increase in Ca^2+^ in the cytoplasm activates CaMKII (calcium/calmodulin-dependent protein kinase II), which promotes apoptosis as an upstream molecule of AMPK [29]. Another key piece of evidence is that endoplasmic reticulum stress induced by 2-deoxyglucose deficiency leads to autophagy by activating AMPK through CaMKK*β* during starvation [30]. Therefore, taking the TM group as a control, we explained the possible key role of the AMPK pathway at the molecular level. On the one hand, the significant difference between the ghrelin and TM cotreatment group and the simple TM group was consistent with the previous experiment; on the other hand, the degree of ERS of the ghrelin-involved group was lower in the two groups pretreated with GSK621, with a decrease in the ROS content and a decrease in the apoptosis rate. However, in the comparison between the ghrelin and TM cotreatment group and the TM alone group, the alleviation effect of ghrelin seemed to be moderated. Whether this can show that ghrelin can play a more effective role when AMPK is activated still requires further experimental verification.

In addition, at the protein level, we can more intuitively see that TM can activate AMPK, and ghrelin can downregulate the phosphorylation level of AMPK and the level of p-AMPK induced by AMPK agonists, which inversely proves the possible relationship between the AMPK pathway and ERS and the important role of ghrelin.

Certainly, we cannot deny previous research results. It has been reported that the protective effect of ghrelin is mediated by stimulating GHSR (growth hormone secretagogue receptor) [31], but the specific downstream mechanism has not been determined. Whether there is a connection needs to be further verified, but the mitigation effect of ghrelin on ERS is positive.

It is worth noting that acylation modification was considered to be an essential factor for the biological activity of ghrelin in the past [32], but in recent years, it has been found that unacylated ghrelin also has certain activity, which may have opposite physiological functions [33] or coordinate with acylated ghrelin to play a regulatory role. In rodent experiments [34], it has been found that unacylated ghrelin can improve vascular function and reduce plaque formation by reducing vascular oxidative stress, but its related receptor and pathway mechanism have not been clearly reported [35]. In addition, there are also “new substances” with different activities or functions by changing the side groups of ghrelin to play the corresponding biological functions, and thus, unveiling their mystery may bring new surprises to the endocrine regulatory system.

## 5. Conclusion

This research may provide new insight into the protective effects of ghrelin on bone by maintaining endoplasmic reticulum homeostasis and regulating of ERS. Ghrelin may alleviate endoplasmic reticulum stress apoptosis induced by tunicamycin in MC3T3E1 cells at least in part by inhibiting AMPK phosphorylation.

## Figures and Tables

**Table 1:** Primers for qRT-PCR.

Gene	Forward primer	Reverse primer
*β*-Actin	5′-ACTGCCGCATCCTCTTCCT-3′	5′-TCAACGTCACACTTCATGATGGA-3′
BIP/GRP78	5′-CTCCGGCGTGAGGTAGAAAA-3′	5′-AGAGCGGAACAGGTCCATGT-3′
CHOP	5′-AGGAGGTCCTGTCCTCAGATGA-3′	5′-ATGTGCGTGTGACCTCTGTTG-3′
IRE1*α*	5′-TGGCCGTTGTAGCTTCAGTCT-3′	5′-CCACCGGAAAGCACGTAATAA-3′

BIP/GRP78, CHOP, and IRE1*α* are all marker genes of endoplasmic reticulum stress, and *β*-actin is a housekeeping gene.

## Data Availability

The data used to support the findings of this study are available from the corresponding authors upon request.

## References

[1] Suzuki R., Fujiwara Y., Saito M. (2020). Intracellular accumulation of advanced glycation end products induces osteoblast apoptosis via endoplasmic reticulum stress. *Journal of bone and mineral research*.

[2] Zheng Z., Shang Y., Tao J., Zhang J., Sha B. (2019). Endoplasmic reticulum stress signaling pathways: activation and diseases. *Current protein & peptide science*.

[3] Yanagi S., Sato T., Kangawa K., Nakazato M. (2018). The homeostatic force of ghrelin. *Cell Metabolism*.

[4] Ding Y., Zhang N., Li J., Jin Y., Shao B. (2018). Molecular cloning and expression of ghrelin in the hypothalamus–pituitary–gastrointestinal tract axis of the Yak (Bos grunniens) in the Qinghai–Tibetan Plateau. *Anatomia, Histologia, Embryologia*.

[5] Ezquerro S., Frühbeck G., Rodríguez A. (2017). Ghrelin and autophagy. *Current opinion in clinical nutrition and metabolic care*.

[6] Ma T., Su Y., Lu S. (2017). Ghrelin expression and significance in 92 patients with atrial fibrillation. *Anatolian journal of cardiology*.

[7] Song B., Yan X. (2021). Ghrelin ameliorates chronic obstructive pulmonary disease-associated infllammation and autophagy. *Biotechnology and Applied Biochemistry*.

[8] Chowen J. A., Argente J. (2017). Ghrelin: a link between energy homeostasis and the immune system. *Endocrinology*.

[9] Rino Y., Aoyama T., Atsumi Y., Yamada T., Yukawa N. (2021). Metabolic bone disorders after gastrectomy: inevitable or preventable?. *Surgery today*.

[10] Napoli N., Pedone C., Pozzilli P. (2011). Effect of ghrelin on bone mass density: the InChianti study. *Bone*.

[11] Herzig S., Shaw R. J. (2018). AMPK: guardian of metabolism and mitochondrial homeostasis. *Nature reviews Molecular cell biology*.

[12] Lin S. C., Hardie D. G. (2018). AMPK: Sensing Glucose as well as Cellular Energy Status. *Cell Metabolism*.

[13] Yang T. L., Shen H., Liu A. (2020). A road map for understanding molecular and genetic determinants of osteoporosis. *Nature Reviews. Endocrinology*.

[14] Yang Y. H., Li B., Zheng X. F. (2014). Oxidative damage to osteoblasts can be alleviated by early autophagy through the endoplasmic reticulum stress pathway--implications for the treatment of osteoporosis. *Free radical biology & medicine*.

[15] Liu W., Zhu X., Wang Q., Wang L. (2013). Hyperglycemia induces endoplasmic reticulum stress-dependent CHOP expression in osteoblasts. *Experimental and therapeutic medicine*.

[16] Yang L., Guan G., Lei L. (2018). Palmitic acid induces human osteoblast-like Saos-2 cell apoptosis via endoplasmic reticulum stress and autophagy. *Cell stress & chaperones*.

[17] Qi Z., Chen L. (2019). Endoplasmic reticulum stress and autophagy. *Advances in experimental medicine and biology*.

[18] Smith M., Wilkinson S. (2017). ER homeostasis and autophagy. *Essays in biochemistry*.

[19] Feng K., Ge Y., Chen Z. (2019). Curcumin inhibits the PERK-eIF2*α*-CHOP pathway through promoting SIRT1 expression in oxidative stress-induced rat chondrocytes and ameliorates osteoarthritis progression in a rat model. *Oxidative Medicine and Cellular Longevity*.

[20] Cao S. S., Kaufman R. J. (2014). Endoplasmic reticulum stress and oxidative stress in cell fate decision and human disease. *Antioxidants & redox signaling*.

[21] Sinha K., Das J., Pal P. B., Sil P. C. (2013). Oxidative stress: the mitochondria-dependent and mitochondria-independent pathways of apoptosis. *Archives of toxicology*.

[22] Xu H., Li Y., Liu R. (2019). Protective effects of ghrelin on brain mitochondria after cardiac arrest and resuscitation. *Neuropeptides*.

[23] Wang X., Wang X. L., Chen H. L. (2014). Ghrelin inhibits doxorubicin cardiotoxicity by inhibiting excessive autophagy through AMPK and p38-MAPK. *Biochemical pharmacology*.

[24] Gumus Balikcioglu P., Balikcioglu M., Muehlbauer M. J. (2015). Macronutrient regulation of ghrelin and peptide YY in pediatric obesity and Prader-Willi syndrome. *The Journal of clinical endocrinology and metabolism*.

[25] Wang Y., Cao L., Liu X. (2019). Ghrelin alleviates endoplasmic reticulum stress and inflammation-mediated reproductive dysfunction induced by stress. *Journal of assisted reproduction and genetics*.

[26] Chung H., Chung H. Y., Bae C. W., Kim C. J., Park S. (2011). Ghrelin suppresses tunicamycin- or thapsigargin-triggered endoplasmic reticulum stress-mediated apoptosis in primary cultured rat cortical neuronal cells. *Endocrine journal*.

[27] Hsu S. K., Chiu C. C., Dahms H. U. (2019). Unfolded protein response (UPR) in survival, dormancy, immunosuppression, metastasis, and treatments of cancer cells. *International Journal of Molecular Sciences*.

[28] Fujiwara Y., Kawaguchi Y., Fujimoto T., Kanayama N., Magari M., Tokumitsu H. (2016). Differential AMPK Recognition of CaMKK Isoforms. *The Journal of biological chemistry*.

[29] Ozcan L., Tabas I. (2010). Pivotal role of calcium/calmodulin-dependent protein kinase II in ER stress-induced apoptosis. *Cell cycle*.

[30] Xi H., Barredo J. C., Merchan J. R., Lampidis T. J. (2013). Endoplasmic reticulum stress induced by 2-deoxyglucose but not glucose starvation activates AMPK through CaMKK*β* leading to autophagy. *Biochemical Pharmacology*.

[31] Wu C. S., Bongmba O. Y. N., Yue J. (2017). Suppression of GHS-R in AgRP neurons mitigates diet-induced obesity by activating thermogenesis. *International journal of molecular sciences*.

[32] Kojima M., Hosoda H., Date Y., Nakazato M., Matsuo H., Kangawa K. (1999). Ghrelin is a growth-hormone-releasing acylated peptide from stomach. *Nature*.

[33] Fernandez G., Cabral A., Cornejo M. P. (2016). Des-acyl ghrelin directly targets the arcuate nucleus in a ghrelin-receptor independent manner and impairs the orexigenic effect of ghrelin. *Journal of Neuroendocrinology*.

[34] Zanetti M., Gortan Cappellari G., Graziani A., Barazzoni R. (2019). Unacylated ghrelin improves vascular dysfunction and attenuates atherosclerosis during high-fat diet consumption in rodents. *International journal of molecular sciences*.

[35] Soares J. B., Leite-Moreira A. F. (2008). Ghrelin, des-acyl ghrelin and obestatin: three pieces of the same puzzle. *Peptides*.

